# Insecticidal Effects of Organotin(IV) Compounds on *Plutella Xylostella* (L.) Larvae. II. Inhibitory Potencies Against Acetylcholinesterase and Evidence for Synergism in Tests With *Bacillus Thuringiensis*(BER.) and Malathion

**DOI:** 10.1155/MBD.1994.1

**Published:** 1994

**Authors:** Nazni W. Ahmad, Tay Siew Huang, S. Balabaskaran, K. M. Lo, V. G. Kumar Das

**Affiliations:** 1 Institute of Advanced Studies University of Malaya Kuala Lumpur 59100 Malaysia; 2 Department of Biochemistry University of Malaya Kuala Lumpur 59100 Malaysia; 3 Department of Chemistry University of Malaya Kuala Lumpur 59100 Malaysia

## Abstract

Features of pesticide synergism and acetylcholinesterase (AChE) inhibition (*in vitro*) were studied using a selected range of organotin compounds against the early 4th instar larvae of a highly resistant strain of the diamondback moth (DBM), *Plutella xylostella*, a major universal pest of cruciferous vegetables.

Fourteen triorganotin compounds were evaluated for their ability to enhance the toxicity of the microbial insecticide, *Bacillus thuringiensis* (BT) and of the commercial insecticide, Malathion to *Plutella xylostella* larvae. Supplemental synergism was observed with triphenyl- and tricyclopentyltin hydroxides in combinations with *Bacillus thuringiensis*. Increased synergism was observed with an increase in the number of cyclopentyl groups on tin in the mixed series, Cyp_n_ Ph_3-n_ SnX, where X = OH, and 1-(1,2,4-triazolyl). The combination of (*p*-chlorophenyl)diphenyltin *N,N*-dimethyldithiocarbamate at LD_10_ and LD_25_ concentrations with sublethal concentrations of Malathion as well as of tricyclohexyltin methanesulphonate at the 0.01% (w/v) concentration with Malathion exerted strong synergistic effects (supplemental synergism) with toxicity index (T.I) values of 7.2, 19.8 and 10.1, respectively.

Studies on the *in vitro* inhibition of acetylcholinesterase prepared from the DBM larvae showed that while most of the triorganotin Compounds tested were without effect on the enzyme, compounds containing the thiocarbamylacetate or the dithiocarbamylacetate moieties demonstrated appreciable levels of inhibition, being comparable in efficacy to commercial grades of Malathion and Methomyl.


### INSECTICIDAL EFFECTS OF ORGANOTIN(IV) COMPOUNDS ON *PLUTELLA XYLOSTELLA* (L.) LARVAE. II. INHIBITORY POTENCIES AGAINST ACETYLCHOLINESTERASE AND EVIDENCE FOR SYNERGISM IN TESTS WITH *BACILLUS THURINGIENSIS* (BER.) AND MALATHION

Nazni W. Ahmad[1], Tay Siew Huang[1], S. Balabaskaran[2], K.M. Lo[1] and V.G. Kumar Das[3]*

[1] Institute of Advanced Studies [2] Department of Biochemistry [3] Department of Chemistry
University of Malaya, 59100 Kuala Lumpur, Malaysia

### ABSTRACT

**Features of pesticide synergism and acetylcholinesterase (AChE) inhibition (*in vitro*) were studied using a selected range of organotin compounds against the early 4th instar larvae of a highly resistant strain of the diamondback moth (DBM), *Plutella xylostella*, a major universal pest of cruciferous vegetables.**

**Fourteen triorganotin compounds were evaluated for their ability to enhance the toxicity of the microbial insecticide, *Bacillus thuringiensis* (BT) and of the commercial insecticide, Malathion to *Plutella xylostella* larvae. Supplemental synergism was observed with triphenyl- and tricyclopentyltin hydroxides in combinations with *Bacillus thuringiensis*. Increased synergism was observed with an increase in the number of cyclopentyl groups on tin in the mixed series, $Cyp_nPh_{3-n}SnX$, where X = OH, and 1-(1,2,4-triazolyl). The combination of (*p*-chlorophenyl)diphenyltin *N,N*-dimethyldithiocarbamate at $LD_{10}$ and $LD_{25}$ concentrations with sublethal concentrations of Malathion as well as of tricyclohexyltin methanesulphonate at the 0.01% (w/v) concentration with Malathion exerted strong synergistic effects (supplemental synergism) with toxicity index (T.I) values of 7.2, 19.8 and 10.1, respectively.**

**Studies on the *in vitro* inhibition of acetylcholinesterase prepared from the DBM larvae showed that while most of the triorganotin compounds tested were without effect on the enzyme, compounds containing the thiocarbamylacetate or the dithiocarbamylacetate moieties demonstrated appreciable levels of inhibition, being comparable in efficacy to commercial grades of Malathion and Methomyl.**

Keywords: Organotins, *Plutella xylostella*, toxicity, synergism, acetylcholinesterase inhibition

---

### INTRODUCTION

Insecticide synergists have considerable practical importance in the control of arthropod pests by "making more efficient the economical use of insecticides, thereby increasing the spectrum of activity of an insecticide and restoring its activity in a resistant strain".[1] The enhancement of the toxic effects of one compound by another or synergism, was first reported in pharmacological studies by Macht.[2] On the other hand, if the combination turns out to be antagonistic, it will result in reduced mortality of the test organism.

---

* Author to whom correspondence should be addressed study. The R-strain was obtained locally from Kea Farm, Cameron Highlands and the larvae were reared in muslin-mesh cages of size 30x30x30 cm, at 28 ± 2°C with a constant relative humidity of 90 ± 6% and a photoperiod of 12:12 (L:D) without exposure to any insecticide. The adults were fed with drops of Holloway medium[4] on cellophane and the larvae were fed on fresh *Brassica chinensis* leaves. For the *in vitro* inhibition studies on AChE, early fourth instar DBM larvae of the susceptible strain obtained from National Chung-Hsiang University, Taichung (Taiwan) were also included.

### *Insecticides*

A total of eighteen organotin compounds, two conventional insecticides [Malathion (Gold Coin$^R$, 84% a.i.) and Methomyl (Lannate$^R$, 90% a.i.)] and one microbial biocide, *Bacillus thuringiensis* Berliner (BT) were used in the toxicological studies. The organotin compounds used in this study were synthesised according to established procedures[26] and were of analytical grade purity. The standard insecticides used were of technical grade quality.

### *Topical bioassay*

Early 4th instar larvae in batches of ten, of average weight 28 ± 3 mg, were lightly anaesthesized with $CO_2$ and treated topically on the dorsal surface by using a Drummond microcap applicator with 1.0 $\mu$Lof test solution. Treatments were carried out at six concentrations for each test compound, while the controls were treated with solvent (acetone) alone. For a complete test, 3 batches of ten larvae each were treated with each of the 6 doses of a given test compound. The treated larvae were then transferred onto fresh *Brassica* leaves and kept at 28 ± 1°C in plastic finger bowls provided with ventilated covers.

Mortality was assessed at 24h and 48h after the topical applications. Larvae that failed to respond to gentle mechanical stimulation were considered dead. The bioassay data were analysed by the Probit Method of Finney[27] to obtain the lethal dosage index values, $LD_{50}$.

### *Determination of BT toxicity*

An aqueous suspension of Thuricide$^R$, a F-Zuelling product containing *Bacillus thuringiensis* Berliner (BT) (1600 IU/mg) as biotic insecticide, together with a wetting agent (Teepol) was applied to both sides of the *Brassica* test leaf.

For each dosage of Thuricide$^R$, the effects on the mortality of ten 4th instar larvae were assessed in triplicate and the mortality data were counted 48h after larval feeding on the treated leaf.

### *Determination of synergistic effects*

In this study two methods for evaluating synergistic effects were employed. In the first method (Method A), a constant sublethal concentration [$LD_{10}$, $LD_{25}$ or 0.01% (w/v)] of the triorganotin compound was applied topically on early 4th instar larvae, pre-starved for 6 hours, and the larvae were then released onto the BT- or Malathion-treated leaves. The Malathion solutions were prepared from the commercial sample of Malathion diluted in distilled water containing a surfactant ('Teepol') to ensure complete wetting of the leaf surface. Four concentrations of BT and Malathion ($LD_{10}$, $LD_{25}$, $LD_{40}$ and $LD_{50}$) were used; for each concentration 100 $\mu$L of solution was applied to the leaf surface. Following air-drying, the leaves were kept with their petioles wrapped in moist cotton wool in plastic finger bowls provided with ventilated covers and at the temperature of 28 ± 1°C. Ten treated larvae were then released onto each leaf. Three replicates were performed for each BT and Malathion concentration.

In the second method (Method B), which more accurately reflects field exposure than the topical application method, both BT or Malathion and the organotin [fixed at a given topical dosage concentration of $LD_{10}$, $LD_{25}$ or 0.01 % (w/v)] were applied as a mixture on the test leaf, prior to the release of the pre-starved (6 h) larvae on its surface. For both bioassays the data procured at the end of 48h were corrected for control mortality using Abbott's formula,[28] and analysed by Finney's probit method[27] to obtain the $LD_{50}$ (or $LC_{50}$) value of the combined BT or Malathion - triorganotin application. The toxicity index[29] (T.I) as defined by the formula below, was calculated.

$$\text{Toxicity Index (T.I.)} = \frac{LD_{50}\ \text{(BT or Malathion)}}{LD_{50}\ \text{(BT or Malathion + Organotin)}}$$

Values of T.I. greater than unity are indicative of synergism, and values less than unity of antagonism.

***In vitro assay of AChE activity and its inhibition by a selected range of triorganotin compounds***

The activity of acetylcholinesterase was measured by the colorimetric method of Ellman[30] using acetylthiocholine iodide (ASChI) as the substrate and 5,5 - dithiobis(2-nitrobenzoic acid) (DTNB), as the reagent. The enzyme activity was measured by following the increase in absorbance at 412 nm arising from the formation of the thionitrobenzoate anion in the enzyme catalysed reaction.

Twenty larvae were homogenized in 30 mL of 0.1 M phosphate buffer (pH 7.0) using a glass homogenizer in an ice-bath. Homogenates were centrifuged for 20 min at 1,500 g. The supernatant was used as the enzyme source.

The reaction mixture consisted of 1 mL enzyme solution, 1.8 mL of 0.1 M phosphate buffer, pH 7.0, 0.1 mL of staining solution (DTNB) and 0.1 mL of substrate (ASChI). The final concentration of substrate and DTNB in the reaction mixture were 0.5 mM and 0.3 mM, respectively. The incubation period was 6 min. Optical density was read on a Bausch & Lomb spectrophotometer at 412 nm against a blank provided by incubating an equivalent volume of the same mixture but omitting the enzyme.

Inhibition studies on the enzyme were performed using 28 triorganotin compounds along with the two commercial insecticides, Malathion and Methomyl, for comparison purposes. A series of six concentrations of the various compounds ($10^{-3}$ - $10^{-8}$ M ) were tested. Three replicates were performed for each assay. From the optical density values, the percentage inhibition was evaluated using the formula:

$$\%\ \text{Inhibition} = \frac{\text{Abs. of control - Abs. of test solution}}{\text{Abs. of control}} \times 100$$

The results obtained were subjected to simple linear regression analysis using the statistical programme (Statsgraphic) to obtain 50% inhibition values ($I_{50}$).

Synergistic combinations require only sub-lethal doses of both chemical and biotic insecticides. Hence synergism has become a favourite topic of research since synergistic combinations not only offer many possibilities in the more potent use of available insecticides, but also, as a result of reduced amounts of chemical insecticides used, check rapid resistance development in the pest. Benz[3] classified synergism on the basis of mortality data into five types, viz. independent, subadditive, supplemental, potentiative and coalitive action synergism.

The diamondback moth, *Plutella xylostella* (L.), represents one of the most notorious cases of control failure caused by insecticide resistance. The high level of resistance of the larvae of this pest to all major groups of chemical insecticides[4-8], i.e. chlorinated hydrocarbons, organophosphates, carbamates, pyrethroids and benzoylphenylureas, as well as microbial insecticides[9], such as the bacterium, *Bacillus thuringiensis* (Ber.), has prompted studies on other control measures such as the use of a synergist to regulate the moth population.

Piperonyl butoxide is a well established synergist which has been used in conjunction with a range of insectides in the control of a number of lepidopterous larvae, but is conspicously ineffective against the DBM larvae.[10] Indeed, no effective synergist has been found todate to overcome insecticide resistance in DBM.[11-13] Applications of insecticides on a rotational basis or their use as mixtures are other possible approaches for overcoming the resistance problem.[14]

Resistance mechanisms in DBM appear to be complex, and relate to several factors such as decreased penetration of insecticides,[15] enhanced detoxification by esterases[16] and glutathione S-transferase,[17,18] and reduced sensitivity of AChE.[5,17,19]

It was therefore of considerable interest to examine a selected range of triorganotin compounds for their potency as synergists when combined with the microbial biocide *Bacillus thuringiensis* and the commercial insecticide Malathion towards the DBM larvae. Fourteen triorganotin compounds were chosen for this purpose based on their acute toxicity data and antifeedant effects.[20]

This paper also includes a study on the effects of organotins on the enzyme acetylcholinesterase (AChE), which is known to be the target molecule in insects for the exertion of lethal effects by the organophosphate and carbamate group of insecticides.[21,22] One way in which insect populations become resistant to these insecticides is by evolution of an altered enzyme.[23,24] Whereas organophosphates and carbamates have been extensively studied as potent AChE inhibitors, there are no reports todate of a systematic screening of triorganotin compounds substituted with organophosphate or carbamate moieties in them as potential inhibitors against this enzyme. This has prompted the additional investigation reported herein of the *in vitro* inhibition of the above enzyme, isolated from resistant strains of *Plutella xylostella* larvae, by a limited range of triorganotin compounds, selected in most cases, on the basis of their high acute larval toxicity. Included among the compounds are several which have previously been shown to be effective inhibitors of glutathione -*S*- transferase,[25] a major detoxifying enzyme present in relatively high levels in resistant strains of DBM larvae.[18]

### MATERIALS AND METHODS

### *Insects*

Early fourth instar larvae of a highly resistant DBM strain were used throughout this

### RESULTS AND DISCUSSION

### *Synergistic effects in tests with BT*

The $LD_{50}$ values for a selected range of triorganotin compounds tested against early 4th instar DBM larvae (R-strain) are given in Table I, and those for the BT - triorganotin mixtures (tested by Methods A and B, see Materials and Methods) in Tables II and III, respectively.

Table I Acute toxicity data[a,b]

| Compound | $LD_{50}$ (g/L) | Molar $LD_{50}$ ($\times 10^{-3}$ moles/L) |
|---|---|---|
| $Ph_3SnX$ | | |
| X = $OC(O)CH_2SC(O)NH(p\text{-}ClC_6H_4)$ | 0.53 ± 0.19 | 0.90 |
| $OCOCH_2SC(O)NHPh$ | 0.85 ± 0.15 | 1.50 |
| $OCOCH_2SC(S)\underline{NCH_2CH_2OCH_2CH_2}$ | 0.94 ± 0.19 | 1.66 |
| $OC(O)CH_2SC(O)NMe_2$ | 0.91 ± 0.31 | 1.80 |
| $OC(O)C_6H_4[NHP(O)(OEt)_2]\text{-}o$ | 1.28 ± 0.53 | 2.06 |
| $OC(O)CH_2SC(S)NMe_2$ | 1.70 ± 0.29 | 3.23 |
| $OC(O)CH_2SC(S)OR'$ (R' = menthyl) | 2.32 ± 0.50 | 3.62 |
| $OC(O)Me$ | 2.47 ± 0.31 | 6.06 |
| $OC(O)CH_2SC(S)OPr^i.H_2O$ | 12.90 ± 0.77 | 24.00 |
| $Cyh_3SnX$ | | |
| X = $OC(O)CH_2SC(S)\underline{NCH_2CH_2CH_2CH_2CH_2}$ | 0.11 ± 0.02 | 0.18 |
| $OC(O)CH_2SC(S)NMe_2$ | 0.81 ± 0.10 | 1.78 |
| $Cyp_3SnX$ | | |
| X = $Cl.Ph_3PO$ | 0.06 ± 0.01 | 0.094 |
| $\underline{NCH{:}NCH{:}N}$ | 0.29 ± 0.02 | 0.27 |
| Cl | 0.12 ± 0.22 | 0.33 |
| OH | 0.36 ± 0.02 | 1.06 |
| $Cyp_2PhSn\underline{NCH{:}NCH{:}N}$ | 0.95 ± 0.05 | 2.37 |
| $Cyp_2PhSnOH$ | 0.95 ± 0.05 | 2.70 |
| $CypPh_2SnOH$ | 1.81 ± 0.16 | 5.04 |
| $CypPh_2Sn\underline{NCH{:}NCH{:}N}$ | 3.22 ± 0.21 | 7.85 |

[a] 4th instar (R-strain); topical application, 48h

[b] Cyh = $c\text{-}C_6H_{11}$ ; Cyp = $c\text{-}C_5H_9$

Table II Computed probit analysis data (48h) obtained for early 4th instar DBM larvae (R-strain) tested against BT in the presence of organotin compounds by Method A*

| Compound | Parameters of Probit line a | b ± S.E | Heterogenity $X^2$ | df | $LC_{50}$ ± S.E (g/L) |
|---|---|---|---|---|---|
| BT alone | 2.75 | 1.11 ± 0.19 | 6.36 | 4 | 0.095 ± 0.023 |
| $Ph_3SnOH$ (1) | 8.15 | 1.51 ± 0.59 | 1.72 | 2 | 0.008 ± 0.001 |
| $Cyp_3SnOH$ (2) | 6.76 | 0.78 ± 0.51 | 2.24 | 2 | 0.006 ± 0.008 |
| $CypPh_2SnOH$ (3) | 5.92 | 0.91 ± 0.53 | 5.09 | 2 | 0.096 ± 0.001 |
| $Cyp_2PhSnOH$ (4) | 6.88 | 1.90 ± 0.63 | 3.77 | 2 | 0.102 ± 0.025 |
| $Cyp_3SnCl$ (5) | 6.62 | 1.56 ± 0.52 | 1.24 | 2 | 0.093 ± 0.023 |
| $Cyp_3SnCl:Ph_3PO$ (6) | 5.94 | 0.85 ± 0.48 | 1.57 | 2 | 0.080 ± 0.003 |
| $Cyp_2PhSnNCH:NCH:N$ (7) | 6.19 | 0.95 ± 0.47 | 1.67 | 2 | 0.056 ± 0.016 |

* The organotins were topically applied on pre-starved larvae at the pre-determined $LD_{25}$ dosage level, and the larvae were then released onto BT-treated test leaves.

Table III Computed probit analysis data (48h) obtained for early 4th instar DBM larvae (R-strain) tested against BT in the presence of organotin compounds by Method B

| Compound | Treatment* | Parameters of Probit line a | b ± S.E | Heterogenity $X^2$ | df | $LC_{50}$ ± S.E (g/L) |
|---|---|---|---|---|---|---|
| BT alone | | 2.75 | 1.11 ± 0.19 | 6.36 | 4 | 0.095 ± 0.023 |
| $Ph_3SnOH$ (1) | $LD_{25}$ | 5.98 | 0.91 ± 0.50 | 2.31 | 2 | 0.084 ± 0.001 |
| | $LD_{10}$ | 6.01 | 0.86 ± 0.47 | 1.88 | 2 | 0.067 ± 0.023 |
| $Cyp_3SnOH$ (2) | $LD_{25}$ | 6.55 | 1.01 ± 0.47 | 4.13 | 2 | 0.033 ± 0.010 |
| | $LD_{10}$ | 6.46 | 1.07 ± 0.47 | 4.02 | 2 | 0.044 ± 0.011 |
| | 0.01%(w/v) | 7.56 | 1.66 ± 0.51 | 0.99 | 2 | 0.029 ± 0.007 |
| $CypPh_2SnOH$ (3) | $LD_{25}$ | 6.31 | 0.97 ± 0.49 | 5.47 | 2 | 0.045 ± 0.010 |
| | $LD_{10}$ | 6.48 | 1.21 ± 0.51 | 6.61 | 1 | 0.059 ± 0.017 |
| | 0.01%(w/v) | 7.12 | 1.93 ± 0.54 | 0.27 | 2 | 0.080 ± 0.014 |
| $Cyp_2PhSnOH$ (4) | $LD_{25}$ | 6.51 | 1.05 ± 0.47 | 1.26 | 2 | 0.036 ± 0.010 |
| | $LD_{10}$ | 5.93 | 0.94 ± 0.49 | 0.34 | 2 | 0.101 ± 0.017 |
| | 0.01%(w/v) | 8.41 | 2.44 ± 0.52 | 2.58 | 2 | 0.040 ± 0.014 |

Cont'd (Table III)

| | | | | | | |
|---|---|---|---|---|---|---|
| $Cyp_3SnCl$ | | | | | | |
| (5) | $LD_{25}$ | 8.02 | 2.72 ± 0.62 | 3.85 | 2 | 0.077 ± 0.001 |
| | $LD_{10}$ | 6.67 | 1.71 ± 0.55 | 0.41 | 2 | 0.105 ± 0.028 |
| $Cyp_3SnCl:Ph_3PO$ | | | | | | |
| (6) | $LD_{25}$ | 6.61 | 1.81 ± 0.59 | 3.26 | 2 | 0.129 ± 0.040 |
| | $LD_{10}$ | 5.76 | 0.96 ± 0.51 | 5.20 | 2 | 0.162 ± 0.101 |
| $Cyp_2PhSnNCH:NCH:N$ | | | | | | |
| (7) | $LD_{25}$ | 7.11 | 1.54 ± 0.48 | 5.40 | 2 | 0.043 ± 0.007 |
| $Cyp_3SnNCH:NCH:N$ | | | | | | |
| (8) | $LD_{25}$ | 7.53 | 1.53 ± 0.50 | 0.40 | 2 | 0.023 ± 0.007 |
| | $LD_{10}$ | 6.05 | 0.91 ± 0.50 | 2.30 | 2 | 0.070 ± 0.024 |
| $CypPh_2SnNCH:NCH:N$ | | | | | | |
| (9) | $LD_{25}$ | 7.17 | 1.63 ± 0.51 | 5.21 | 2 | 0.047 ± 0.008 |
| | $LD_{10}$ | 6.39 | 1.32 ± 0.53 | 4.19 | 2 | 0.089 ± 0.026 |

* Organotin at the fixed topical dosage concentration indicated was applied as a mixture with BT on the test leaf; four BT concentrations ($LD_{10}$, $LD_{25}$, $LD_{40}$ and $LD_{50}$) were used for this purpose.

As determined by Method A, supplemental synergism[3] is clearly indicated for triphenyltin hydroxide. For sublethal BT concentrations of $LD_{10}$, $LD_{25}$, $LD_{40}$ and $LD_{50}$ on the leaf, this organotin compound applied at the $LD_{25}$ level on the larvae led to percentage mortalities of 77, 80, 93 and 97, respectively (Table IV), which are far greater than the algebraic sum of the single effects of BT and the organotin. Tricyclopentyltin hydroxide similarly exhibited supplemental synergism with percentage mortalities of 70, 73, 73 and 90, respectively. Replacement of one or more of the cyclopentyl groups in tricyclopentyltin hydroxide by phenyl reduced the synergistic effect.

In Method B, the organotin (at either the $LD_{10}$ or $LD_{25}$ topical dosage level), although of a concentration not sufficient to cause larval mortality on the leaf, however, proved highly active in combinations with BT, leading to mortalities higher than expected on the basis of the sublethal concentrations of BT used. For tricyclopentyltin hydroxide, the toxicity index is about 3 at the $LD_{25}$ and 0.01%(w/v) dosage levels and 2 at the $LD_{10}$ level, whereas a higher toxicity index of 17 (Table V) is obtained when the test is performed by Method A. However, in practical applications in the field where the larvae are found on the leaves, the use of tricyclopentyltin - BT mixtures at the $LD_{25}$ level may be anticipated to yield a better performance than that indicated by the data in Method B.

As was the case noted from the data procured by Method A, the results from Method B also substantiate the increased synergism observed with increasing replacement of the phenyl groups by cyclopentyl groups in the series $Cyp_nPh_{3-n}SnX$ [X = OH, 1-(1,2,4-triazolyl)].

Table IV Percentage mortality (48h), assessed by Method A, of early 4th instar DBM larvae exposed to Malathion in the presence of triorganotin compounds[a]

| Malathion (g/L) | $M_A$[b] (on leaf) (%) | $M_S$[b] (topical) (%) | $M_{A+S}$ (%) (1) | (2) | (3) | (4) | (5) | (6) | (7) |
|---|---|---|---|---|---|---|---|---|---|
| 0.021 | 10 | 25 | 76.7 | 70.0 | 33.3 | 10.0 | 16.7 | 26.7 | 36.7 |
| 0.045 | 25 | 25 | 80.0 | 73.3 | 40.0 | 33.3 | 30.0 | 50.0 | 43.3 |
| 0.071 | 40 | 25 | 93.3 | 73.3 | 50.0 | 40.0 | 40.0 | 50.0 | 50.0 |
| 0.095 | 50 | 25 | 96.7 | 90.0 | 53.3 | 46.7 | 53.3 | 46.7 | 63.3 |

[a] Compounds: (1) Triphenyltin hydroxide; (2) Tricyclopentyltin hydroxide; (3) Diphenylcyclopentyltin hydroxide; (4) Dicyclopentylphenyltin hydroxide; (5) Tricyclopentyltin chloride; (6) Tricyclopentyltin chloride.Triphenylphosphine oxide(1:1 adduct); (7) Phenyldicyclopentyltin (1,2,4-triazole); (8) Tricyclopentyltin (1,2,4-triazole); (9) Diphenylcyclopentyltin (1,2,4-triazole)

[b] $M_A$ = % mortality due to BT; $M_S$ = % mortality due to organotin compound

Table V Toxicity of BT in the presence of triorganotin compounds (48h) to early fourth instar DBM larvae[a]

| Concentration (g/L) | Method A | Method B | | |
|---|---|---|---|---|
| Organotin[b] compound | $LD_{50}$ of organotin-BT mixture $LD_{25}$[c] | $LD_{50}$ of organotin-BT mixture $LD_{10}$[c] | $LD_{25}$[c] | 0.01%(w/v)[c] |
| (1) | 0.008 (11.6) | 0.067 (1.4) | 0.084 (1.1) | n.d. |
| (2) | 0.006 (16.9) | 0.044 (2.2) | 0.033 (2.9) | 0.029 (3.3) |
| (3) | 0.096 (1.0) | 0.059 (1.6) | 0.045 (2.1) | 0.080 (1.2) |
| (4) | 0.102 (0.9) | 0.101 (0.9) | 0.036 (2.6) | 0.040 (2.2) |
| (5) | 0.093 (1.0) | 0.105 (0.9) | 0.077 (1.2) | n.d. |
| (6) | 0.080 (1.2) | 0.162 (0.6) | 0.129 (0.7) | n.d. |
| (7) | 0.056 (1.7) | n.d. | 0.043 (2.2) | n.d. |

Cont'd (Table V)

| | | | | |
|---|---|---|---|---|
| (8) | n.d. | 0.070 (1.3) | 0.023 (4.2) | n.d. |
| (9) | n.d. | 0.089 (1.1) | 0.047 (2.0) | n.d. |

[a] Calculated values of the toxicity index are given in parentheses
[b] See Table IV for identity of compounds
[c] Fixed concentration of organotin
n.d: not determined

***Synergistic effects in tests with Malathion***

The $LC_{50}$ values obtained for the Malathion - triorganotin mixtures in tests against the R - strain of DBM larvae based on both Methods A and B are given in Tables VI and VII, respectively.

It is seen, in general, that the $LD_{50}$ values of the Malathion-organotin mixtures are lower in the cases for which the organotin component was mixed at the $LD_{25}$ concentration. Based on their intrinsic $LD_{25}$ values, the relative toxicity trend of the organotin compounds are (13) > (12) > (11) > (10) > (14), but in their binary mixtures with Malathion at the $LD_{50}$ concentration, the most active mixture was obtained with compound (14) for each of Methods A and B. It was found that Method B appeared to elicit a better response on the larvae inasmuch as the $LC_{50}$ values for all the compounds were consistently lower than the corresponding values obtained by Method A. Based on Method A, the order of activity of the mixtures (with the organotin at the $LD_{25}$ concentration) as discerned from their $LC_{50}$ values is:

(14) > (13) > (10) ≈ (12) > (11)

while the order with the organotin at the 0.01%(w/v) concentration is:

(13) > (12) > (14) > (10) > (11)

For Method B, the corresponding activity trends are respectively,

(14) > (10) > (11) > (13) > (12) and

(13) > (12) ≈ (14) >> (11) >> (10).

The percentage mortality data assessed by Method A are tabulated in Table VIII. For all the organotin compounds mortality values obtained were less than the algebraic sum of the single effects of Malathion and the specific organotin, but the values were, however, greater than the computed mortalities based on independent synergism (*vide supra*). This result is characteristic of subadditive synergism, and is corroborated also by the data in Table IX based on Method B. Of the compounds studied by either method, (*p*-chlorophenyl)diphenyltin *N,N*-dimethyldithiocarbamatoacetate (compound 14) proved to be most effective as a synergist. As shown in Table IX mixtures of Malathion at $LC_{10}$, $LC_{25}$,

Table VI Computed probit analysis data (48h) obtained for early 4th instar DBM larvae (R-strain) tested against Malathion in the presence of organotin compounds by Method A

| Compound | Treatment* | Parameters of Probit line a | b ± S.E | Heterogenity $X^2$ | df | $LC_{50}$ ± S.E (g/L) |
|---|---|---|---|---|---|---|
| $Ph_3SnOC(O)(CH_2)_2C(O)Ph$ | | | | | | |
| | $LD_{10}$: 0.32 | 2.78 | 1.75 ± 0.52 | 0.55 | 2 | 18.32 ± 4.36 |
| (10) | $LD_{25}$: 0.67 | 3.35 | 1.48 ± 0.48 | 0.15 | 2 | 13.10 ± 2.75 |
| | 0.01%(w/v) | 3.25 | 1.29 ± 0.50 | 0.10 | 2 | 22.46 ± 8.60 |
| $Ph_3SnOC(O)CH_2SSnPh_3$ | | | | | | |
| | $LD_{10}$: 0.06 | 3.26 | 1.31 ± 0.50 | 0.66 | 2 | 21.36 ± 7.76 |
| (11) | $LD_{25}$: 0.20 | 3.14 | 1.51 ± 0.49 | 1.55 | 2 | 17.29 ± 4.51 |
| | 0.01%(w/v) | 2.87 | 1.54 ± 0.53 | 0.91 | 2 | 24.03 ± 8.37 |
| $Cyh_3SnSC_5H_4N\text{->}O$ | | | | | | |
| | $LD_{10}$: 0.007 | 3.57 | 0.98 ± 0.49 | 0.92 | 2 | 29.25 ± 18.15 |
| (12) | $LD_{25}$: 0.02 | 3.97 | 0.91 ± 0.46 | 0.45 | 2 | 13.41 ± 4.61 |
| | 0.01%(w/v) | 4.20 | 1.01 ± 0.46 | 0.97 | 2 | 6.17 ± 1.96 |
| $Cyh_3SnO_3SMe$ | | | | | | |
| | $LD_{10}$: 0.003 | 3.60 | 0.10 ± 0.48 | 1.19 | 2 | 25.61 ± 14.03 |
| (13) | $LD_{25}$: 0.012 | 3.71 | 1.20 ± 0.47 | 0.42 | 2 | 11.82 ± 2.84 |
| | 0.01%(w/v) | 4.89 | 0.74 ± 0.48 | 0.03 | 2 | 1.40 ± 1.73 |
| $(p\text{-}ClC_6H_4)Ph_2SnX$ ($X = OC(O)CH_2SC(S)NMe_2$) | | | | | | |
| | $LD_{10}$: 0.33 | 2.71 | 1.53 ± 0.57 | 0.65 | 2 | 31.12 ± 13.84 |
| (14) | $LD_{25}$: 0.85 | 4.72 | 0.49 ± 0.45 | 0.01 | 2 | 3.76 ± 3.66 |
| | 0.01%(w/v) | 3.50 | 1.21 ± 0.48 | 0.38 | 2 | 17.30 ± 5.58 |

* The organotins were topically applied on pre- starved larvas at the pre- determined $LD_{25}$, $LD_{10}$ and 0.01%(w/v) dosage levels and the larvae were then released onto Malathion-treated leaves.

Table VII Computed probit analysis data (48h) obtained for early 4th instar DBM larvae (R-strain) tested against Malathion in the presence of organotin compounds by Method B

| Compound | Treatment* | Parameters of Probit line a | b ± S.E | Heterogenity $X^2$ | df | $LC_{50}$ ± S.E (g/L) |
|---|---|---|---|---|---|---|
| $Ph_3SnOC(O)(CH_2)_2C(O)Ph$ | | | | | | |
| | $LD_{10}$: 0.32 | 3.03 | 1.38 ± 0.52 | 0.33 | 2 | 26.48 ± 11.09 |
| (10) | $LD_{25}$: 0.67 | 4.45 | 0.63 ± 0.45 | 0.01 | 2 | 7.27 ± 3.26 |
| | 0.01%(w/v) | 3.26 | 0.93 ± 0.53 | 0.81 | 2 | 71.30 ± 33.62 |
| $Ph_3SnOC(O)CH_2SSnPh_3$ | | | | | | |
| | $LD_{10}$: 0.06 | 3.04 | 1.60 ± 0.50 | 0.24 | 2 | 16.78 ± 4.01 |
| (11) | $LD_{25}$: 0.20 | 4.31 | 0.76 ± 0.45 | 0.01 | 2 | 8.07 ± 2.88 |
| | 0.01%(w/v) | 3.61 | 0.91 ± 0.49 | 0.06 | 2 | 33.59 ± 24.63 |
| $Cyh_3SnSC_5H_4N\text{->}O$ | | | | | | |
| | $LD_{10}$: 0.007 | 3.58 | 0.91 ± 0.49 | 0.54 | 2 | 35.80 ± 27.51 |
| (12) | $LD_{25}$: 0.02 | 3.21 | 1.54 ± 0.48 | 0.49 | 2 | 14.31 ± 3.08 |
| | 0.01%(w/v) | 4.02 | 0.95 ± 0.46 | 0.88 | 2 | 10.73 ± 3.10 |
| $Cyh_3SnO_3SMe$ | | | | | | |
| | $LD_{10}$: 0.003 | 3.52 | 1.23 ± 0.48 | 0.09 | 2 | 15.76 ± 4.61 |
| (13) | $LD_{25}$: 0.01 | 3.99 | 0.96 ± 0.46 | 0.17 | 2 | 11.29 ± 3.30 |
| | 0.01%(w/v) | 4.10 | 1.36 ± 0.47 | 2.44 | 2 | 4.60 ± 1.38 |

Cont'd (Table VII)

| | | | | | | |
|---|---|---|---|---|---|---|
| ($p$-$ClC_6H_4$)$Ph_2SnX$ (X = OC(O)$CH_2$SC(S)$NMe_2$) | | | | | | |
| | $LD_{10}$: 0.33 | 4.86 | 0.48 ± 0.45 | 0.17 | 2 | 1.95 ± 3.06 |
| (14) | $LD_{25}$: 0.85 | 5.09 | 0.59 ± 0.48 | 0.01 | 2 | 0.71 ± 0.14 |
| | 0.01%(w/v) | 3.96 | 1.03 ± 0.46 | 0.42 | 2 | 10.11 ± 2.63 |

Organotin at the fixed topical dosage concentration indicated was applied as a mixture with Malathion on the test leaf; four Malathion concentrations ($LD_{10}$, $LD_{25}$, $LD_{40}$ and $LD_{60}$) were used for this purpose.

Table VIII Percentage mortality (48h), assessed by Method A, of early 4th instar DBM larvae exposed to Malathion in the presence of triorganotin compounds [a]

| Malathion (g/L) | $M_B$[b] (on leaf) (%) | $M_S$[b] (topical) (%) | $M_{B+S}$ (%) (10) | (11) | (12) | (13) | (14) |
|---|---|---|---|---|---|---|---|
| 3.810 | 17.24 | 25 | 20.0 | 20.0 | 33.3 | 30.0 | 50.0 |
| 7.873 | 27.60 | 25 | 40.0 | 23.3 | 36.7 | 36.7 | 56.7 |
| 13.182 | 41.40 | 25 | 50.0 | 40.0 | 50.0 | 53.3 | 60.0 |
| 17.873 | 51.70 | 25 | 56.7 | 56.7 | 60.0 | 60.0 | 63.3 |

[a] Compounds:(10) Triphenyltin 3-benzoylpropionate; (11) *O,S*-bis(tri-phenyltin)mercapto-acetate; (12) Tricyclohexyltin(2-pyridinethiolato-*N*-oxide); (13) Tricyclohexyltin-methane-sulphonate; (14) ($p$-chlorophenyl)diphenyltin *N,N*- dimethyldithiocarbamatoacetate

[b] $M_B$ = % mortality due to Malathion; $M_S$ = % mortality due to organotin compound

Table IX Percentage mortality (48h), assessed by Method B, of early 4th instar DBM larvae exposed to Malathion in the presence of triorganotin compounds [a]

| Organotin compound (S): | (10) | (11) | (12) | (14) |
|---|---|---|---|---|
| $M_S$[b] on leaf (%) | (21.4) | (17.9) | (10.7) | (28.6) |
| $M_B$ on leaf (%) | $M_{B+S}$ on leaf (%) | | | |
| 17.2 | 43.3 | 40.0 | 20.0 | 66.7 |
| 27.6 | 50.0 | 50.0 | 33.3 | 73.3 |
| 41.4 | 56.7 | 56.7 | 43.3 | 76.6 |
| 51.7 | 60.0 | 63.3 | 56.7 | 80.0 |

[a] See footnotes to Table VIII; [b] Applied topically at the $LD_{25}$ dosage level

$LC_{40}$ or $LC_{50}$ concentrations with this compound at the $LD_{25}$ concentration level led to larval mortalities on the leaf of 66.7, 73.3, 76.7 and 80.0 percent, respectively. Inasmuch as the mortality values are significantly higher than for the other cases, it is conceivable that supplemental rather than subadditive synergism might be operative with compound (14). One possible reason for the increased efficacy of compound (14) is that it contains the dithiocarbamato ligand fragment, which latter is known to be insecticidally active in its own right.[31]

Computations of Toxicity Index (T.I.) values (Table X) reveal a high T.I. (19.8) for compound (14) at the sublethal $LD_{25}$ concentration as assessed by Method B; the corresponding value as obtained by Method A is 3.8. Inasmuch as the results are based on tests conducted on resistant larvae, the potential of compound (14) as a synergist in application with Malathion has obvious commercial appeal. Only one other compound, namely tricyclohexyltin methanesulphonate, gave T.I. value of 3.1 as assessed by Method B. The apparent high T.I. of this compound could be due to the high dosage [0.01%(w/v)] thus causing significant larval mortality (c.f. topical $LD_{50}$ dosage = 0.0549 g $L^{-1}$)[20]. In general, however, Method B which is more effective and practical in field studies than Method A allowed a better assessment of synergistic effects of organotin compounds when used with Malathion.

It is to be noted that no larval mortality occurred at the end of 24h in the presence of organotin alone when applied on leaf at $LD_{25}$ topical dosage concentration, whereas with Malathion, mortality was evident even at the end of 24h. Thus, in studies using Method B mortality was recorded at the end of 48h. The possibility that larval mortality recorded at the end of 48h could also be induced, at least partially, by starvation as a result of antifeedant effects exerted by the organotin compound under test cannot, of course, be excluded.

Table X Toxicity of Malathion in the presence of triorganotin compounds (48h) to early fourth instar DBM larvae[a]

| Concentration (g/L) | Method A | | | Method B | | |
|---|---|---|---|---|---|---|
| | $LD_{50}$ of organotin-Malathion mixture | | | $LD_{50}$ of organotin-Malathion mixture | | |
| Organotin[b] compound | $LD_{10}$[c] | $LD_{25}$[c] | 0.01%[c](w/v) | $LD_{10}$[c] | $LD_{25}$[c] | 0.01%[c](w/v) |
| (10) | 18.322 **(0.8)** | 13.107 **(1.1)** | 22.462 **(0.6)** | 26.481 **(0.5)** | 7.279 **(1.9)** | 71.304 **(0.2)** |
| (11) | 21.366 **(0.7)** | 17.290 **(0.8)** | 24.030 **(0.6)** | 16.785 **(0.8)** | 8.066 **(1.8)** | 33.592 **(0.4)** |
| (12) | 29.246 **(0.5)** | 13.410 **(1.05)** | 6.174 **(2.3)** | 35.797 **(0.4)** | 14.318 **(1.0)** | 10.792 **(1.3)** |
| (13) | 25.607 **(0.6)** | 11.823 **(1.19)** | 1.397 **(10.09)** | 15.765 **(0.9)** | 11.287 **(1.3)** | 4.601 **(3.1)** |
| (14) | 31.115 **(0.5)** | 3.761 **(3.8)** | 17.304 **(0.8)** | 1.949 **(7.2)** | 0.710 **(19.8)** | 10.114 **(1.4)** |

[a] Calculated values of the toxicity index are given in parentheses; [b] See Table VIII for identity of compounds; [c] Fixed concentration of organotin

It is, however, worth noting that compound (14) which showed high T.I. value when used at the ($LD_{25}$) sublethal concentration with Malathion, exhibited only a modest antifeedant effect in contrast with the other organotin compounds.[20]

The above result along with evidence for synergism with *Bacillus thuringiensis* or Malathion presented herein suggests that the same series of compounds may also prove to be effective in combination with other classes of chemical or microbial insecticides. Further work in this direction should prove rewarding, including especially a biochemical study enquiring into the mode of synergistic action.

### *In vitro inhibition studies on AChE*

The cholinesterases are a group of enzymes of which acetylcholinesterase (AChE) is the most significant because of its role in the regulation of nervous impulses across neuron/neuron and neuron/target tissue synapses. In such a system, an action potential arriving at the distal end of an axon causes the release of the neurotransmitter acetylcholine which diffuses across the synapse to activate the cholinergic receptors of another neuron or the target tissue. As the enzyme AChE hydrolyzes acetylcholine, synaptic transmission ceases and the nerve membranes return to their resting potential and are prepared for the next stimulus.[32]

AChE has important toxicological significance because it is readily inhibited by phosphate and carbamate esters that are commonly used as insecticides. The inhibition causes nervous transmission to continue unchecked and results in a neuromuscular malfunction which can be lethal.[21,22]

The potential of triorganotin compounds to inhibit acetylcholinesterase has been sparsely explored in the literature, the best documented work being that of Blum and Bower[33] who reported that triethyltin hydroxide was capable of causing rapid paralysis of house flies (*Musca domestica*) (L.) upon topical application. Triethyltin carboxylate esters, however, failed to inhibit cholinesterase but proved effective in blocking conduction in the isolated central nerve cord of the American cockroach, *Periplaneta americana* (L.).[33]

In the present study, some twenty five selected triorganotin compounds were screened for their anticholinesterase activity using *in vitro* preparations of the enzyme derived from resistant and susceptible strains of DBM larvae. The results are presented in Tables XI and XII, where the $I_{50}$ dose, expressed in moles $L^{-1}$, is the concentration of the test compounds that reduces the activity of the AChE to half the control level. In the majority of cases, the compounds are totally ineffective against the enzyme, with estimated $I_{50}$ values well exceeding $10^6$ moles $L^{-1}$. Noteworthy exceptions were provided by the compounds containing the thiocarbamyl and dithiocarbamyl moiety in the ester residue. Triphenyltin *N,N*-dimethylthiocarbamyl acetate, $Ph_3SnOC(O)CH_2SC(O)NMe_2$, is seen to be $10^3$-fold more active than the corresponding dithiocarbamylacetate, $Ph_3SnOC(O)CH_2SC(S)NMe_2$, but suprisingly a dramatic drop (over $10^6$-fold) in activity attends the replacement of a tin-bound phenyl group in $Ph_3SnOC(O)CH_2SC(S)NMe_2$ by a *p*-chlorophenyl group (Table XI). This trend is not reflected in the results from tests conducted on the enzyme derived from the suceptible strain of the DBM larvae, where a $10^2$-fold increase in activity is noted for ($p$-$ClC_6H_4)Ph_2SnOC(O)CH_2SC(S)NMe_2$ over the triphenyltin analogue (Table XII). This probably hints of an altered structure for AChE in the resistant strain, but more work is necessary to establish this point.

Table XI Relationship between $LD_{50}$ and $I_{50}$ values obtained for selected triorganotin compounds and commercial insecticides in tests against 4th instar DBM larvae

| Inhibitor | $LD_{50}$ moles/L | $I_{50}$ moles/L |
|---|---|---|
| $Ph_3SnOC(O)C(O)Me$ | $4.50 \times 10^{-3}$ | $1.22 \times 10^{5}$ |
| $Ph_3SnOC(O)(CH_2)_2C(O)Me$ | $6.95 \times 10^{-3}$ | a |
| $Ph_3SnOC(O)(CH_2)_2\ C(O)Ph$ | $1.84 \times 10^{-3}$ | a |
| $Ph_3SnOC(O)(CH_2)_2C(:NNHC(O)NH_2)Me$ | $2.22 \times 10^{-3}$ | a |
| $Cyh_3SnOCOCH_2SC(S)NMe_2$ | $1.78 \times 10^{-3}$ | a |
| $Cyh_3SnOC(O)CH_2SC(S)NCH_2CH_2CH_2CH_2CH_2$ | $0.22 \times 10^{-3}$ | a |
| $Cyh_3SnOC(O)CH_2(8\text{-}C_9H_6NO)$ | $0.57 \times 10^{-3}$ | a |
| $Ph_3SnOC(O)CH_2OC_6H_4Cl\text{-}p$ | $2.26 \times 10^{-3}$ | $7.07 \times 10^{2}$ |
| $Ph_3SnOC(O)CH_2OC_6H_4NO_2\text{-}p$ | $4.38 \times 10^{-3}$ | a |
| $Ph_3SnOC(O)CH_2OC_6H_4COOH\text{-}p$ | $0.69 \times 10^{-3}$ | $1.30 \times 10^{2}$ |
| $Ph_3SnSC:NN:C(NH_2)S$ | $2.31 \times 10^{-3}$ | a |
| $Ph_3SnOC(O)CH_2SC(S)NMe_2$ | $3.23 \times 10^{-3}$ | 16.91 |
| $Ph_3SnOC(O)CH_2SCSNCH_2CH_2OCH_2CH_2$ | $1.66 \times 10^{-3}$ | 2.50 |
| $Ph_3SnOC(O)CH_2SC(O)NMe_2$ | $1.80 \times 10^{-3}$ | $1.31 \times 10^{-2}$ |
| $(p\text{-}ClC_6H_4)Ph_2SnOC(O)CH_2SC(S)NMe_2$ | $2.36 \times 10^{-3}$ | $3.01 \times 10^{6}$ |
| $Ph_3SnOC(O)CH_2SC(O)NH(p\text{-}ClC_6H_4)$ | $0.90 \times 10^{-3}$ | $9.0 \times 10^{-5}$ |
| $Ph_3SnOC(O)C_6H_4[NHP(O)(OEt_2)]\text{-}o$ | $2.06 \times 10^{-3}$ | 2.77 |
| Malathion | $42.6 \times 10^{-3}$ | 5.69 |
| Methomyl | $1.43 \times 10^{-3}$ | $2.36 \times 10^{-3}$ |

[a] Estimated values are in excess of $10^6$ moles $L^{-1}$

Table XII Data concentration values of organotins for 50% inhibition of AChE derived from R- and S-strains of 4th instar DBM larvae

| Inhibitor | Values of $I_{50}$ (moles/L) | |
|---|---|---|
| | R-strain | S-strain |
| $Ph_3SnOC(O)CH_2OC_6H_4COOH\text{-}p$ | $1.30 \times 10^{2}$ | 5.920 |
| $Ph_3SnOC(O)CH_2SC(S)NMe_2$ | $1.69 \times 10^{1}$ | $2.62 \times 10^{-2}$ |
| $Ph_3SnOC(O)CH_2SC(O)NMe_2$ | $1.31 \times 10^{-2}$ | $5.43 \times 10^{-6}$ |
| $(p\text{-}ClC_6H_4)Ph_2SnOC(O)CH_2SC(S)NMe_2$ | $3.01 \times 10^{6}$ | $2.40 \times 10^{-4}$ |
| $Ph_3SnOC(O)C_6H_4[NHP(O)(OEt_2)]\text{-}o$ | 2.77 | $7.89 \times 10^{-1}$ |
| Malathion | 5.69 | $3.32 \times 10^{-2}$ |
| Methomyl | $2.36 \times 10^{-3}$ | $3.51 \times 10^{-4}$ |

$I_{50}$ = Inhibitor concentration causing 50% inhibiton of enzyme activity

The acute toxicity data ($LD_{50}$ values) for the dithiocarbamylacetates are given in Table I. It is interesting to note that the dithiocarbamylacetate, $Ph_3SnOC(O)CH_2SC(S)NMe_2$ is more toxic than $Ph_3SnOAc$; replacement of the thione sulphur by oxygen further improves the activity, but replacement of the dialkylamino group by an alkoxide (xanthylacetate) results in a siginificant lowering of activity. Within both the dithiocarbamatoacetates and xanthylacetates, influences of remote substituents on activity are apparent. Thus, $Ph_3SnOC(O)CH_2SC(S)NMe_2$ is less toxic than $Ph_3SnOC(O)CH_2SC(S)NCH_2CH_2OCH_2CH_2$ and $Ph_3SnOCOCH_2SC(S)OPr^{(i)}.H_2O$ less toxic than $Ph_3SnOC(O)CH_2SC(S)OR'$ (R' = menthyl). Among the monothiocarbamates, no significant differences in activity are encountered upon changing the organic groups bound to nitrogen.

A focussed study on the inhibition of AChE derived from the resistant strain of the DBM larvae was attempted using a range of triphenylstannylthiocarbamyl acetates at the $10^{-4}$M concentration. The percentage inhibition data (Table XIII) clearly attest to the superior inhibitory potency of the compound $Ph_3SnOCOCH_2SC(O)NH(\textit{p}\text{-}ClC_6H_4)$, containing the *p*-chlorophenyl group attached to nitrogen. Placement of two chlorine substituents in the amido phenyl ring reduces the activity markedly from 74.6% in the above compound to 13.4% in $Ph_3SnOC(O)CH_2SC(O)NH(\textit{o,p}\text{-}Cl_2C_6H_3)$.

The lowered activity of $(\textit{p}\text{-}ClC_6H_4)Ph_2SnOC(O)CH_2SC(O)NHPh$ relative to the triphenyltin analogue is again apparent from the percentage inhibition data (15.81% *vs* 31.12%), although the difference is not as marked as that encountered with the dithiocarbamylacetates.

Returning to Table XI, it is apparent that the following organotin compounds have an inhibitory potency comparable to that of commercial grades of Malathion ($I_{50}$ 5.69M) and Methomyl ($I_{50}$ 2.4 x $10^{-3}$M) used in the tests:

| | |
|---|---|
| $Ph_3SnOC(O)CH_2SC(O)NMe_2$ | ($I_{50}$: 1.3x$10^{-2}$M), |
| $Ph_3SnOC(O)CH_2SC(S)NCH_2CH_2OCH_2CH_2$ | ($I_{50}$: 2.50M), |
| $Ph_3SnOC(O)C_6H_4$ [*o*-NHP(O)(OEt)$_2$] | ($I_{50}$: 2.8M), |
| $Ph_3SnOC(O)CH_2SC(O)NH(\textit{p}\text{-}ClC_6H_4)$ | ($I_{50}$: 9.0x$10^{-5}$M), |

However, no correlation is evident between the $I_{50}$ and the $LD_{50}$ values for the entire range of compounds investigated. As seen from Table XI, the molar $I_{50}$ values of the compounds are subtantially higher than the molar $LD_{50}$ values, except for the compounds, triphenyltin *N*-*p*-chlorophenyl monothiocarbamatoacetate and Methomyl. Little can be said at this point as to the reasons for the seemingly better competitivenes of these two compounds for the AChE binding sites in the presence of the substrate, ASChI. Clearly, a wider range of stannylated monothiocarbamates as well as carbamates need to be studied with a view to identifying the roles played by the carbamyl (thiocarbamyl) moiety as much as by the triorganostannyl moiety.

Table XIII Percentage inhibition of AChE (derived from 4th instar resistant-strain DBM larvae) in *in vitro* tests using $10^{-4}$M concentration of triorganotins

| Compound | % inhibition |
|---|---|
| $Ph_3SnOC(O)CH_2SC(O)NH(c\text{-}C_6H_{11})$ | 27.55 |
| $Ph_3SnOC(O)CH_2SC(O)NHPh$ | 31.12 |
| $Ph_3SnOC(O)CH_2SC(O)NH(p\text{-}ClC_6H_4)$ | 74.60 |
| $Ph_3SnOC(O)CH_2SC(O)NH(o,p\text{-}Cl_2C_6H_3)$ | 13.40 |
| $(p\text{-}ClC_6H_4)Ph_2SnOC(O)CH_2SC(O)NHPh$ | 15.81 |
| $Ph_3SnOC(O)CH_2SC(O)NCH_2CH_2CH_2CH_2CH_2$ | 24.37 |
| $Ph_3SnOC(O)(CH_2)_5SC(O)NCH_2CH_2CH_2CH_2CH_2$ | 27.03 |
| $Ph_3SnSC(O)NCH_2CH_2CH_2CH_2CH_2$ | 14.81 |
| $Ph_3SnOC(O)CH_2SC(O)NMe_2$ | 42.10 |
| $Ph_3SnOC(O)CH_2SC(S)NMe_2$ | 31.77 |

### ACKNOWLEDGEMENTS

We are indebted to the National Science Council for Research and Development, Malaysia (Grant No. 2-07-04-06) for generous support of this work. One of us (Nazni W.A.) is grateful to the University of Malaya for a Postgraduate Fellowship Award. We thank Professor C.N. Sun, National Chung-Hsiang University, Taichung (Taiwan) for the generous donation of a stock of the susceptible strain of DBM, and Dr. Mohd. Sofian-Azirun, Department of Zoology, University of Malaya, for the commercial sample of Methomyl.

### REFERENCES

1. Metcalf, R L *Ann. Rev. Entomol.*, 1967, **12**: 229
2. Macht, E *Drug Metabol. Dispos.*, 1929, **1**: 391
3. Benz, G Synergism of microorganisms and chemical insecticides. In: *Microbial Control of Insects and Mites*, Burgess, H D and Hussey, N W (eds), Academic Press, New York, 1971, pp 327-555
4. Sudderuddin, K I and Kok, P F *FAO Plant Proc. Bull.*, 1978, **26**: 53
5. Sun, C N, Wu, T K, Chen, J S and Lee, W T Insecticide resistance in diamondback moth management. In: *Proceedings International Workshop on Diamondback Moth Management,* 11-15 March 1985, AVDRC, Shanhua, Taiwan, pp 359-371
6. Liu, M Y, Tzeng, Y J and Sun, C N *J. Econ. Entomol.,*1982, **75**: 153
7. Abro, G H, Dybas, R A, Green, AST.J and Wright, D J *J.Econ. Entomol.,* 1988, **81**: 1575
8. Cheng, E Y *Pestic. Sci.,* 1988, **23**: 177
9. Tabashnik, B E, Cushing, N L, Finson, N and Johson, M W *J. Econ. Entomol.,* 1990, **83** (85): 1671
10. Chen, J S and Sun, C N *J. Econ. Entomol.*, 1986, **78**: 22
11. Liu, M Y, Tzeng, Y J and Sun, C N *J. Econ. Entomol.*, 1981, **74**: 393

12. Miyata, T, Saito, T and Noppun, V Studies on the mechanism of diamondback moth resistance to insecticides. In: *Proceedings International Workshop on Diamondback Moth Management,* 11-15 March 1985, AVRDC, Shanhua, Taiwan, pp 347-357
13. Noppun, V, Miyata, T and Saito, T Cross-resistance spectrum and joint toxic action in phenthoate selected strains of the diamondback moth (*Plutella xylostella* L.) *Annual Meeting of the Japanese Society Applied Entomology and Zoology,* 2-4 April 1984, Utsunomiya, Abstract, pp 74
14. Georghiou, G P, Lagunes, A and Baker J D Effect of insecticide rotation on evolution of resistance. In: *IUPAC Pesticide Chemistry, Human Welfare and Environment. 3. Mode of Action, Metabolism and Toxicology,* Matsunaka, S, Hudson, D H and Murphy, S D (eds),Pergamon Press, New York, 1983 pp 183-189
15. Noppun, V, Miyata, T and Saito, T *Appl. Entomol. Zool.*, 1987, **22**: 116
16. Maa, W C and Chuang, M L *Bull. Inst. Zool. Acad. Sin.*, 1983, **22**: 123
17. Wu, T K *M. Sc. Thesis, National Chung Hsiang University, Taichung, Taiwan, Republic of China,* 1983
18. Cheng, E Y, Chou, T M and Kao, C H *J. Agric. Res. China*, 1983, **32**: 373
19. Hama, H *Appl. Entomol. Zool.*, 1987, **22**: 116
20. Nazni, W A, Sofian A M, Balabaskaran, S and Kumar Das, V G *Appl. Organomet. Chem.,* in press.
21. Corbett, J R *The Biochemical Mode of Action of Pesticides,* Academic Press, N.Y, 1974, Chap. 3, pp 107-164
22. O'Brien, R D Acetylcholinesterase and its inhibition. In: *Insecticide Biochemistry and Physiology,* Wilkinson, C F (ed), Plenum Press, New York, 1976 pp 271-295
23. Oppenoorth, F J and Welling, W Biochemistry and Physiology of Resistance. In: *Insecticide Biochemistry and Physiology,* Wilkinson, C F (ed), Plenum Press, New York, 1976 pp 507-551
24. Plapp, F W *Ann. Rev. Entomol.*, 1976, **21**: 179
25. Balabaskaran, S, Uma Devi, P, Seow, S C and Kumar Das, V G Purification of GSH-S-transferase from *Plutella xylostella* larvae and its inhibition by organotin compounds. In: *Chemistry and Technology of Silicon and Tin,* Kumar Das V G, Ng S W and Marcel Gielen, (eds) Oxford University Press, Oxford, 1992, pp 273-288
26. Dub, M *Organometallic Compounds,* Vol.**2** (2nd. Edn.), Springer-Verlag, N.Y., 1967, pp 441-818
27. Finney, D J *Probit Analysis,* (3rd. Edition), Cambridge University Press, London, 1971: 318
28. Abbott, W S *J. Econ. Entomol.*, 1925, **18**: 265
29. Sun, Y P and Johnson, E R *J. Econ. Entomol.*, 1960, **53**: 887
30. Ellman, G L, Courtney, K D, Andres, V Jr. and Featherstone, R M *Biochem. Pharmacol.*, 1961, **7**: 88
31. Kumar Das, V G, Kuthubutheen, A J, Balabaskaran, S and Ng, S W *Main Group Metal Chem.,* 1989, **12**: 389
32. Silver, A *The Biology of Cholinesterase,* North Holland, American Elsevier, 1974
33. Blum, M S and Bower, F A *J. Econ. Entomol.*, 1957, **50**: 84

**Received: April 16, 1993 - Accepted: May 24, 1993 - Received in revised camera-ready form: July 1, 1993**

